# Trabecular bone score may indicate chronic kidney disease-mineral and bone disorder (CKD-MBD) phenotypes in hemodialysis patients: a prospective observational study

**DOI:** 10.1186/s12882-020-01944-0

**Published:** 2020-07-25

**Authors:** Hyo Jin Yun, Soo Ryeong Ryoo, Jung-Eun Kim, Yong Jun Choi, Inwhee Park, Gyu-Tae Shin, Heungsoo Kim, Jong Cheol Jeong

**Affiliations:** 1 Department of Nephrology, Ajou University School of Medicine, Suwon, South Korea; 2 Department of Endocrinology and Metabolism, Ajou University School of Medicine, Suwon, South Korea; 3 Department of Internal medicine, Seoul National University Bundang Hospital, Seongnam, South Korea

**Keywords:** Trabecular bone score, End stage renal disease, Hemodialysis, Chronic kidney disease-mineral and bone disorder, Fracture, Cardiovascular events, Mortality

## Abstract

**Background:**

In the general population, the trabecular bone score (TBS) represents the bone microarchitecture and predicts fracture risk independent of bone mineral density (BMD). A few studies reported that TBS is significantly reduced in dialysis patients. Chronic kidney disease-mineral and bone disorder (CKD-MBD) are accompanied by increased fracture risk, cardiovascular morbidity, and mortality. We investigated whether TBS is associated with comorbidity related to CKD-MBD or frailty in hemodialysis patients.

**Methods:**

In this prospective observational study, TBS was obtained using the TBS iNsight software program (Med-Imaps) with BMD dual energy x-ray absorptiometry (DXA) images (L1–L4) from prevalent hemodialysis patients. A Tilburg frailty indicator was used to evaluate frailty, and hand grip strength and bio-impedance (InBody) were measured. A patient-generated subjective global assessment (PG-SGA) was used for nutritional assessment. The history of cardiovascular events (CVE) and demographic, clinical, laboratory, and biomarker data were collated. We then followed up patients for the occurrence of CKD-MBD related complications.

**Results:**

We enrolled 57 patients in total. The mean age was 56.8 ± 15.9 years (50.9% female). Prevalence of Diabetes mellitus (DM) was 40.4% and CVE was 36.8%. Mean TBS was 1.44 ± 0.10. TBS significantly reduced in the CVE group (1.38 ± 0.08 vs. 1.48 ± 0.10, *p* <  0.001). Multivariable regression analysis was conducted adjusting for age, sex, dialysis vintage, DM, CVE, albumin, intact parathyroid hormone, fibroblast growth factor 23, handgrip strength, and phosphate binder dose. Age (**ß** = − 0.030; *p* = 0.001) and CVE (**ß** = − 0.055; *p* = 0.024) were significant predictors of TBS. During the follow up period after TBS measurements (about 20 months), four deaths, seven incident fractures, and six new onset CVE were recorded. Lower TBS was associated with mortality (*p* = 0.049) or new onset fracture (*p* = 0.007, by log-rank test).

**Conclusion:**

Lower TBS was independently associated with increased age and CVE prevalence in hemodialysis patients. Mortality and fracture incidence were significantly higher in patients with lower TBS values. These findings suggest that TBS may indicate a phenotype of frailty and also a CKD-MBD phenotype reciprocal to CVE.

## Background

Chronic kidney disease-mineral and bone disorder (CKD-MBD) is a systemic disorder that manifests with laboratory and bone abnormalities, and vascular or soft tissue calcification and is associated with an increased risk of fracture, cardiovascular disease, and mortality [1]. In dialysis patients, cardiovascular mortality is 10 to 20 times higher [2], and the relative risk of hip fracture is about four times higher than in the general population [3]. The prevalence of vertebral fracture is more than 50% in hemodialysis patients [4]*.* As a result, frailty and other clinical outcomes are common in chronic kidney disease (CKD) patients.

While the 2017 Kidney Disease Improving Global Outcomes (KDIGO) guidelines advocate bone mineral density (BMD) testing to assess fracture risk in CKD-MBD patients [5], BMD is less predictive of fracture in dialysis patients than in the general population [6, 7]. A reason for this is the overestimation of BMD in CKD patients due to arthritic conditions, scoliosis of the lumbar spine, and the presence of vascular or joint calcifications, which are all common in advanced CKD [8]. In addition, BMD only measures bone quantity, providing no information on trabecular microarchitecture or components of bone quality, which are also important for bone strength in CKD patients [1]. Bone biopsy and imaging methods, such as high-resolution peripheral quantitative computed tomography (HR-pQCT) and micro-magnetic resonance imaging (MRI), can measure bone microarchitecture and, thus, indicate fracture risk; however, their high cost, invasiveness, and low availability limit their routine clinical application.

Trabecular bone score (TBS) is a recently developed diagnostic tool for assessing the image texture obtained from standard lumbar spine dual-energy x-ray absorptiometry (DXA) and provides information on bone microarchitecture independent of BMD [9]. Higher TBS values represent more homogenous, strong, and fracture-resistant bone. TBS can simply be derived from the available DXA images and requires no additional scanning time or radiation exposure. Moreover, TBS is inexpensive, non-invasive, and readily clinically available compared to non-DXA imaging or bone biopsy [10, 11]. Previous studies have demonstrated that TBS correlates with both cortical and trabecular 3-dimensional microarchitecture parameters, such as trabecular volume, number, thickness, spacing, connectivity, and stiffness measured by HR-pQCT or micro-CT [12–15].

To date, only a few studies have assessed TBS in end stage renal disease (ESRD) patients [16–20]. The studies have shown that TBS was significantly reduced in ESRD populations [17, 19, 20], and lower TBS is associated with increased prevalent or incident fracture in CKD patients [11, 18]. However, no studies have assessed the connections between TBS and adverse clinical outcomes related to CKD-MBD in hemodialysis patients, except those studying fracture events.

In this study, we investigated whether TBS is associated with the comorbidities related to CKD-MBD or frailty in hemodialysis patients.

## Methods

### Study design and population

We performed a single-center, prospective, observational study with data from prevalent hemodialysis patients aged over 19 years. Data were collected between May and June, 2016. Exclusion criteria were a history of liver cirrhosis, the presence of a pacemaker, receiving current chemotherapy due to malignancy, pregnancy, and uncooperative behavior due to a psychiatric disorder. After enrollment, we reviewed the medical records for demographic and clinical data, checked laboratory data, and measured frailty index and TBS. We then followed up patients for the occurrence of CKD-MBD-related complications, such as all-cause mortality, incident fracture, and new-onset cardiovascular events (CVE). CVE was defined as a composite of coronary artery disease (CAD), stroke, and peripheral arterial occlusive disease (PAOD).

The study protocol was reviewed and approved by the ethics committee of the Ajou University Hospital (IRB No: AJIRB-MED-SUR-16-128). The study was conducted in accordance with the declaration of Helsinki, and all participants provided their written informed consent.

### Data collection

#### Medical records

We collected demographics and clinical data, such as patient’s age, gender, dialysis vintage, body mass index (BMI), and past medical history, including diabetes mellitus (DM), hypertension (HTN), and CVE, from previous medical records. We also gathered medication history, including phosphate binders, vitamin D metabolites, calcimimetics, warfarin, and proton pump inhibitors (PPI).

#### Serum biochemistry and biomarkers

Fasting blood samples were taken via vascular access (arteriovenous fistula, graft, or tunneled cuffed dialysis catheter), just before the hemodialysis session at the time of BMD and TBS measurement and were stored below − 20 °C until subsequent assays. We measured biochemical parameters associated with bone metabolism, including serum calcium, phosphate, intact parathyroid hormone (PTH), and total alkaline phosphatase (ALP). Intact PTH was measured with an electrochemiluminescence immunoassay (ECLIA) (Cobas, Roche Diagnostic GmbH, Mannheim, Germany). We also measured fibroblast growth factor 23 (FGF 23) and α-klotho concentrations. FGF 23 was measured using a single-plex assay with the R-PLEX Human FGF-23 Antibody set (Meso scale discovery, MD, USA) and α-klotho by an enzyme-linked immunosorbent assay (ELISA), using the human soluble α-Klotho assay kit (Immuno-Biological Laboratories, Gunma, Japan).

#### Frailty and nutritional assessment

For frailty assessment, we used the Tilburg frailty indicator, a standardized self-reporting questionnaire about physical, psychologic, and social aspects of individual functioning. For nutritional assessment, we used various methods. As to body composition, we performed bioelectrical impedance analysis (BIA) using a multifrequency bio-impedance device (InBody S10, InBody Co. Ltd., Seoul, Korea), according to manufacturer’s instruction. As a functional test, handgrip strength was measured on the non-fistula side with a portable Jamar plus Digital Hand Dynamometer (Sammons Preston Rolyan, Bolingbrook, IL, USA). We also measured serum albumin, blood urea nitrogen (BUN), creatinine, and potassium. Nutritional assessment was completed using the Patient-Generated Subjective Global Assessment (PG-SGA), nutritional scoring system which consists of medical history and physical examination components.

#### Trabecular bone score

All participants had a BMD measurement using DXA (Lunar Prodigy, GE Lunar, Madison, WI, USA) at the lumbar spine (L1–L4). All measurements were performed by experienced operators using the same machine and standardized procedures. TBS was assessed using TBS iNsight software (version 2.1, Med-Imaps, Pessac, France) with a DXA image and calculated as the mean value of L1–L4. Some vertebrae that were unsuitable for study (due to compression fracture, degenerative change, or any other reasons) were excluded from TBS assessment.

### Statistical analysis

All statistical analyses were performed with STATA statistical software, version 12.1 (StataCorp LP, College Station, Texas, USA). Data are expressed as the mean ± standard deviation (SD) for continuous variables and proportions for categorical variables. Continuous data were evaluated for normality before statistical testing. We used a T-test for normally distributed continuous data and a Mann-Whitney test for non-normally distributed continuous data. For categorical variables, the Chi-square test or Fisher’s exact test were used. Linear regression models and multivariable regression analyses adjusted for relevant covariates were used to investigate predictors of TBS. We conducted Kaplan-Meier time-to-event analysis with a log-rank test and a cox regression test for all-cause mortality, incident fracture, and new onset CVE. A *p*-value < 0.05 was considered statistically significant.

## Results

### Patient’s baseline characteristics

Demographic, clinical, and laboratory characteristics of the patients, including parameters of frailty and DXA, are presented in Table 1. A total of 57 patients were enrolled (49.1% male) with a mean age of 56.8 ± 15.9 years. The average dialysis vintage was 5.9 ± 4.9 years. Patient medical histories included 23 patients (40.4%) with DM, 51 (89.5%) with HTN, 18 (31.6%) with CAD, 5 (8.8%) with stroke, and 4 (7.0%) with PAOD. Eleven patients showed prevalent vertebral fracture. The mean TBS value was 1.44 ± 0.10.

**Table 1:** Patients’ baseline characteristics

Variable	Total (*N* = 57)	CVE (*N* = 21)	No CVE (*N* = 36)	*p*-value
**Demographic characteristics**				
Age (years)	56.8 ± 15.9	65.1 ± 12.4	52.0 ± 15.9	0.002
Male	28 (49.1%)	9 (42.8%)	19 (52.8%)	0.470
Dialysis vintage (years)	5.9 ± 4.9	6.3 ± 4.2	5.7 ± 5.3	0.625
Body mass index (kg/m^2^)	23.1 ± 3.6	23.9 ± 4.4	22.7 ± 3.0	0.230
**Comorbid conditions**				
Diabetes mellitus	23 (40.4%)	11 (52.4%)	12 (33.3%)	0.157
Hypertension^†^	51 (89.5%)	19 (90.5%)	32 (88.9%)	1.000
Coronary artery disease	18 (31.6%)	18 (85.7%)	–	
Stroke	5 (8.8%)	5 (23.8%)	–	
Peripheral arterial disease	4 (7.0%)	4 (19.0%)	–	
**Laboratory measurements**				
Hemoglobin (g/dl)	10.3 ± 0.9	10.5 ± 0.9	10.2 ± 0.8	0.281
Blood urea nitrogen (mg/dl)	65.6 ± 15.1	60.0 ± 14.6	68.9 ± 14.5	0.031
Creatinine (mg/dl)	10.3 ± 3.5	9.5 ± 3.8	10.7 ± 3.3	0.252
Sodium (mmol/L) ^*^	137.2 ± 3.2	136.7 ± 3.1	137.6 ± 3.3	0.484
Potassium (mmol/L)	5.2 ± 0.7	5.1 ± 0.8	5.2 ± 0.6	0.594
Albumin (g/dl) ^*^	3.8 ± 0.5	3.7 ± 0.4	3.9 ± 0.5	0.004
Calcium (mg/dl)	9.1 ± 0.8	9.1 ± 0.7	9.1 ± 0.8	0.806
Phosphate (mg/dl)	4.9 ± 1.5	4.4 ± 1.4	5.2 ± 1.5	0.049
Alkaline phosphatase (U/L)	79.4 ± 72.6	79.1 ± 33.0	79.6 ± 88.4	0.980
Intact PTH (pg/ml) ^*^	282.6 ± 245.8	237.9 ± 201.2	308.8 ± 267.7	0.447
Triglyceride (mg/dl) ^*^	97.8 ± 65.1	100.1 ± 64.4	96.4 ± 66.4	0.591
Low density lipoprotein (mg/dl)	79.1 ± 28.1	77.6 ± 30.7	80.0 ± 26.8	0.755
α-klotho (pg/ml) ^*^	542.9 ± 627.7	474.6 ± 370.0	582.8 ± 740.1	0.381
FGF 23 (pg/ml) ^*^	7185.7 ± 10,737.0	4628.1 ± 8915.9	8677.6 ± 11,524.5	0.077
**Frailty and nutritional assessment**				
Tilburg frailty indicator^*^	3.8 ± 2.6	4.7 ± 3.3	3.2 ± 2.0	0.121
Handgrip strength (kg.f) ^*^	21.7 ± 12.0	18.0 ± 11.9	23.9 ± 11.6	0.031
Phase angle (°)	5.0 ± 1.4	4.5 ± 1.5	5.2 ± 1.2	0.045
PG-SGA^*^	3.9 ± 5.2	5.5 ± 7.1	2.9 ± 3.4	0.095
**Dual-energy x-ray absorptiometry parameters and TBS**				
L-spine BMD (g/cm^2^) ^*^	1.019 ± 0.221	0.943 ± 0.255	1.062 ± 0.188	0.017
T-score (L1–L4) ^*^	−1.2 ± 1.8	−1.8 ± 2.1	− 0.8 ± 1.6	0.021
Trabecular bone score	1.44 ± 0.10	1.38 ± 0.08	1.48 ± 0.10	< 0.001
**Medication use**				
Phosphate binder^†^	52 (91.2%)	17 (81.0%)	35 (97.2%)	0.056
Vitamin D analogue	37 (64.9%)	11 (52.4%)	26 (72.2%)	0.130
Cinacalcet^†^	4 (7.0%)	0 (0.0%)	4 (11.1%)	0.285
Proton pump inhibitor^†^	11 (19.3%)	7 (33.3%)	4 (11.1%)	0.078
Warfarin^†^	1 (1.8%)	1 (4.8%)	0 (0.0%)	0.368

A t-test was used for continuous variables and the χ^2^ test was used for categorical variables except ^*^ and ^†^

*Abbreviations*: *CVE* Cardiovascular events, *PTH* Parathyroid hormone, *FGF 23* Fibroblast growth factor 23, *PG-SGA* Patient-generated subjective global assessment, *BMD* Bone mineral density

^*^p-value by Mann–Whitney *U* test

^†^*p*-value by Fisher’s exact test

After the study population was divided into two groups according to their past history of CVE, CVE was prevalent in 21 patients (36.8%). The age of the CVE group was significantly higher than the no CVE group (65.1 ± 12.4 vs. 52.0 ± 15.9, *p* = 0.002). TBS was significantly reduced in CVE group (1.38 ± 0.08 vs. 1.48 ± 0.10, *p* <  0.001).

### Comparison of TBS according to comorbid conditions, age, and frailty index

**Fig. 1:**
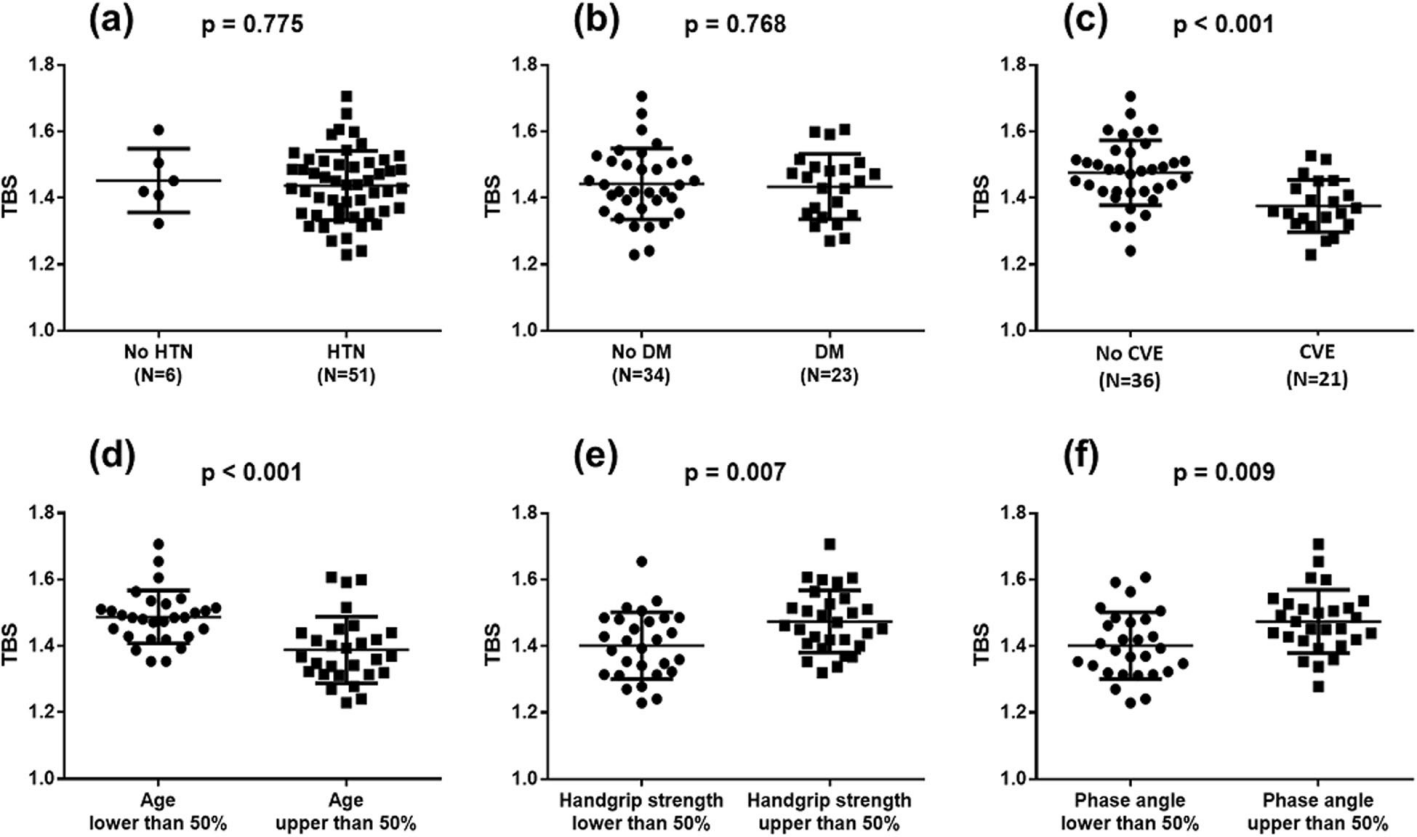
Comparison of Trabecular bone score (TBS) according to comorbid conditions, age, and frailty index. **a** Hypertension (HTN), **b** Diabetes mellitus (DM), **c** Cardiovascular events (CVE), **d** Age, **e** Handgrip strength, and **f** Phase angle

When divided according to each comorbid condition, unlike CVE, TBS did not show any significant differences between patients with or without DM or HTN. Patients above the median age showed significantly lower TBS (*p* <  0.001). Also, patients below the median handgrip strength (*p* = 0.007) and phase angle (*p* = 0.009) values showed significantly lower TBS compared to those above (Fig. 1).

### Factors associated with TBS in hemodialysis patients

To identify the factors associated with TBS, we performed linear regression analysis (Table 2). In univariate analysis, TBS significantly and inversely correlated with age (ß = − 0.042, *p* <  0.001), and positively correlated with creatinine (ß = 0.009, *p* = 0.019) and intact PTH (ß = 0.011, *p* = 0.039). As in previous description, TBS was inversely associated with history of CVE (ß = − 0.010, *p* < 0.001). A significant association was also found between TBS and the parameters related to frailty. Hand grip strength (*r* = 0.039, *p* < 0.001) and phase angle (*r* = 0.030, *p* = 0.002) positively correlated, and PG-SGA (*r* = − 0.006, *p* = 0.029) inversely correlated with TBS. BMD was strongly associated with TBS with a regression coefficient of 0.185 (*p* = 0.002). FGF 23 and α-klotho did not show any significant association with TBS.

**Table 2:** Univariable and multivariable regression analysis of factors associated with trabecular bone score in hemodialysis patients

Variable (*N* = 57)	Univariable			Multivariable		
	ß	95% CI	*p*-value	ß	95% CI	*p*-value^†^
Age, per 10 years	−0.042	−0.055 – −0.028	< 0.001	− 0.030	− 0.049 – − 0.012	0.001
Female vs. Male	− 0.051	− 0.105 – 0.002	0.059	− 0.012	−0.084 – 0.061	0.747
Dialysis vintage (years)	−0.002	−0.008 – 0.003	0.426	0.001	−0.004 – 0.006	0.702
BMI, per 5 kg/m^2^	−0.024	−0.058 – 0.010	0.162			
DM vs. Non-DM	−0.008	−0.064 – 0.048	0.768	0.034	−0.017 – 0.086	0.186
CVE vs. No-CVE	−0.010	−0.150 – − 0.050	< 0.001	−0.055	− 0.103 – − 0.008	0.024
Hemoglobin (g/dl)	0.016	−0.017 – 0.048	0.333			
BUN, per 10 mg/dl	0.016	−0.001 – 0.034	0.071			
Creatinine (mg/dl)	0.009	0.002–0.017	0.019			
Albumin (g/dl)	0.043	−0.016 – 0.103	0.146	0.001	−0.051 – 0.054	0.957
LDL, per 50 mg/dl	0.024	−0.021 – 0.068	0.294			
Calcium (mg/dl)	−0.031	−0.067 – 0.006	0.096			
Phosphate (mg/dl)	0.017	−0.001 – 0.034	0.061			
ALP, per 25 U/L	0.007	−0.003 – 0.016	0.152			
iPTH, per 100 pg/ml	0.011	0.001–0.022	0.039	0.005	−0.005 – 0.015	0.321
ln α-klotho (pg/ml)	0.007	−0.047 – 0.062	0.793			
ln FGF 23 (pg/ml)	0.008	−0.009 – 0.025	0.334	−0.004	−0.020 – 0.012	0.594
Tilburg frailty indicator	−0.003	−0.013 – 0.008	0.626			
Handgrip strength, per 10 kg.f	0.039	0.019–0.059	< 0.001	0.020	−0.012 – 0.051	0.213
Phase angle (°)	0.030	0.011–0.048	0.002			
PG-SGA	−0.006	− 0.011 – 0.001	0.029			
Phosphate binder dose^a^	0.012	−0.003 – 0.029	0.114	−0.001	−0.016 – 0.015	0.919
L-spine BMD (g/cm^2^)	0.185	0.069–0.301	0.002			

*Abbreviations*: ß, Regression coefficient of univariable or multivariable linear regression, *CI* Confidence interval, BMI, Body mass index, *DM* Diabetes mellitus, *CVE* Cardiovascular events, *BUN* Blood urea nitrogen, *LDL* Low density lipoprotein, *ALP* Alkaline phosphatase, *PTH* Parathyroid hormone, *FGF 23* Fibroblast growth factor 23, *PG-SGA* Patient-generated subjective global assessment, *BMD* Bone mineral density

^a^Phosphate binder dose (per BSA/week), per 3500 mg

^†^Adjusted *p*-value

Multivariable regression analysis was conducted adjusting for age, sex, dialysis vintage, DM, CVE, albumin, FGF 23, intact PTH, handgrip strength, and phosphate binder dose. Age (**ß** = − 0.030; *p* = 0.001) and CVE (**ß** = − 0.055; *p* = 0.024) were significant and independent predictors of TBS. There was no significant association between FGF 23 and TBS (*p* = 0.594).

### TBS and clinical outcomes associated with CKD-MBD

We followed-up individuals for an average of 20 months. During follow-up, 4 (7.0%) patients died and there were 7 (12.8%) incident fracture events and 6 (10.5%) new-onset CVE. Among 7 cases of fractures, there were 1 axial bone fracture (vertebra), and 6 appendicular bone fractures (1 hip fracture, 2 other lower extremities, and 3 upper extremities). TBS was significantly lower in the mortality group than the survival group (1.33 ± 0.04 vs. 1.45 ± 0.10; *p* = 0.016). TBS was also lower in patients who had incident fractures than those who did not (1.32 ± 0.05 vs. 1.46 ± 0.10; *p* < 0.001).

**Fig. 2:**
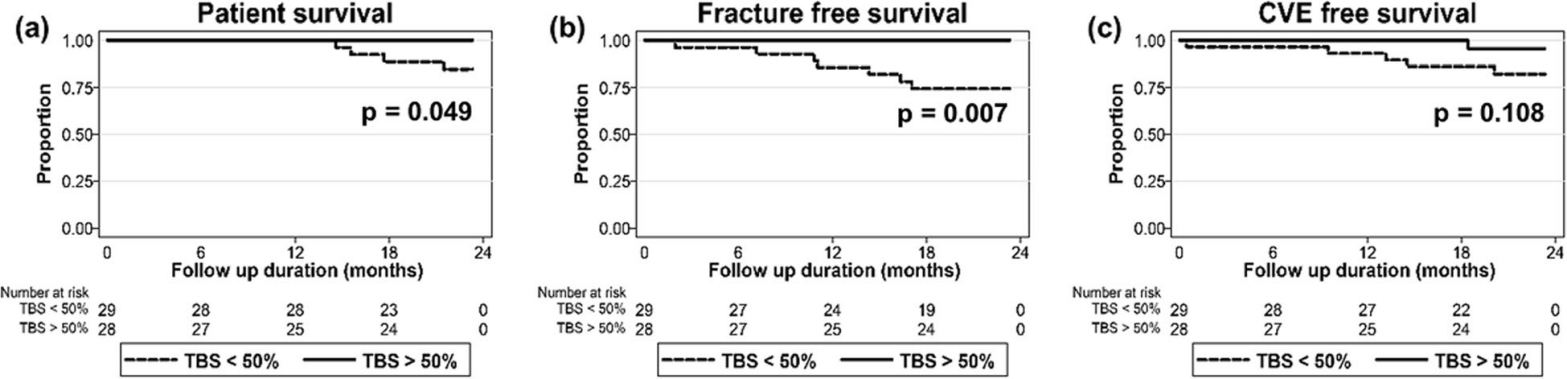
Kaplan-Meier curve stratified by Trabecular bone score (TBS) median. **a** Mortality, **b** Incident fracture, and **c** Cardiovascular events (CVE)

We compared 20-month all-cause mortality, incident fracture, and new-onset CVE probability in patients with TBS values below versus above median. Individuals who had a TBS value below median had a significantly higher probability of mortality (*p* = 0.049) and incident fracture (*p* = 0.007) than those with a value above median (Fig. 2). New-onset CVE tended to be more frequent in patients with TBS values below median, although this did not achieve statistical significance (*p* = 0.108). There were no significant differences in mortality, incident fracture, and new-onset CVE between the patient group with above and below median BMD values (data not shown).

Table 3 shows the results of Cox regression analysis to evaluate the association between TBS and adverse outcomes related to CKD-MBD in the hemodialysis patients. TBS significantly predicted incident fracture with an unadjusted hazard ratio (per 0.1 higher TBS) 0.220 (95% Confidence interval 0.085–0.566, *p* = 0.002). The incident fracture hazard ratio per 0.1 higher TBS was also significant, even after adjustment with several relevant covariates. However, TBS did not show any significance as a predictor of mortality or new onset CVE.

**Table 3:** Hazard ratios for TBS to predict adverse outcomes related to CKD-MBD in hemodialysis patients

Adjustment	Mortality		Incident fracture		New onset CVE	
	HR^a^ (95% CI)	*p*-value	HR^a^ (95% CI)	*p*-value	HR^a^ (95% CI)	*p*-value
Unadjusted	0.314 (0.098–1.004)	0.051	0.220 (0.085–0.566)	0.002	0.450 (0.188–1.075)	0.072
Age	0.640 (0.150–2.725)	0.546	0.209 (0.062–0.700)	0.011	0.573 (0.186–1.770)	0.333
Model 1	0.630 (0.141–2.826)	0.546	0.165 (0.055–0.500)	0.001	0.647 (0.208–2.014)	0.452
Model 2	0.506 (0.116–2.198)	0.363	0.084 (0.014–0.519)	0.008	0.389 (0.107–1.418)	0.153
Model 3	0.369 (0.054–2.492)	0.306	0.041 (0.003–0.488)	0.012	0.403 (0.114–1.430)	0.160
Model 4	0.362 (0.044–2.956)	0.343	0.064 (0.006–0.682)	0.023	0.334 (0.077–1.442)	0.142

Model 1: adjusted for age, dialysis vintage, BMI

Model 2: adjusted for model 1 plus DM, CVE

Model 3: adjusted for model 2 plus PTH

Model 4: adjusted for model 3 plus hand grip test

*Abbreviations*: *CI* Confidence interval, *CKD-MBD* Chronic kidney disease-mineral and bone disorder, *HR* Hazard ratio, *CVE* Cardiovascular events, *TBS* Trabecular bone score

^a^Hazard ratios per 0.1 higher TBS

## Discussion

In this study, we found that old age and prevalent CVE were independently related to lower TBS. Individuals with lower TBS values had significantly higher mortality and incident fracture events. These results suggest that TBS is associated with frailty and comorbidity related to CKD-MBD. To the best of our knowledge, this is the first study to evaluate the relationships between TBS and CKD-MBD related complications, including mortality and cardiovascular disease, in hemodialysis patients.

Vascular calcification (VC), especially coronary artery calcification (CAC), is important for cardiovascular disease, which is the leading cause of death in patients with CKD. Although we did not measure vascular calcification, we demonstrated that lower TBS is associated with prevalent CVE and all-cause mortality in hemodialysis patients. VC has complex pathophysiologic mechanisms and has various traditional and non-traditional risk factors. One of the non-traditional risk factors prevalent in ESRD patient is impaired bone mineral metabolism, possibly due to so-called bone-vascular axis [21], in which abnormal bone turnover and remodeling lead to increased bone resorption or a decreased capacity of bone to buffer calcium and phosphate, resulting in movement of calcium from bone to vessels. It has already been shown that bone microarchitecture, as assessed by HR-pQCT, is inversely associated with CAC [22], and improvement in bone turnover alleviates CAC progression in hemodialysis patients [23]. TBS was reported to be inversely related to abdominal aortic calcification in dialysis patients, although BMD did not show any relationship [24].

Lower TBS is associated with an increased risk of fracture in CKD patients [11, 18]. Similar to prior studies, we showed that participants with a below median TBS had a significantly higher incidence of fracture events. There are several reasons for the increased risk of fracture in dialysis patients, besides renal osteodystrophy; sarcopenia, disability, malnutrition, comorbidities, polypharmacy, autonomic dysfunction, peripheral neuropathy etc. in hemodialysis patients increase their risk of falling [25, 26], which in turn leads to increased fracture risk and frailty. There is accumulating evidence of a link between impaired muscle status and poor bone health. Bone loss and sarcopenia share common pathways, and both are common in CKD patients. Bone and muscle interact not only through mechanical effects but also biochemical communication [27]. In our study, phase angle and hand grip strength, which represent muscle mass, muscle function, and nutritional status, showed a significant positive correlation with TBS. One previous cross-sectional study revealed that the TBS values of older people were significantly lower in women with low grip strength and men with low physical performance [28].

TBS is correlated with BMD not only in the general population but also in CKD patients [16–20]. As in previous studies, our data show there was a significant positive correlation between TBS and lumbar spine BMD. But, unlike TBS, lower BMD was not associated with mortality or incident fracture events in this study. A recent study demonstrated that TBS reflects trabecular microarchitecture measured by bone biopsy in patients with CKD [12]. Therefore, our results suggest that bone microarchitecture (bone quality) may have a greater impact on the clinical outcome than bone volume (quantity) in ESRD patients. Trabecular bone has a higher surface area, thus, a greater capacity for mineral buffering than cortical bone. Therefore, loss of trabecular bone may be a more sensitive representation of adverse outcomes such as fracture or vascular calcification.

FGF 23 is a hormone secreted by osteocytes and osteoblasts and is, potentially, a key initiating biomarker for CKD-MBD, as it increases early in the course of CKD and can be 1000-fold higher than the normal range in advanced CKD [29]. The main target of FGF 23 is the kidneys, where it increases phosphorus excretion and inhibits vitamin D. α-klotho is co-receptor for FGF 23, and is downregulated in CKD patients which results in many adverse outcomes, such as abnormal mineralization or cardiovascular disease [30]. FGF 23 also regulates bone metabolism, demonstrating both over- and under- expression resulting in impaired bone mineralization. Several previous studies found that FGF 23 is not associated with bone mass, as assessed by BMD in hemodialysis patients [31, 32], whereas α-klotho positively correlated with BMD [32]. However, a recent study reported that FGF 23 levels negatively correlate with bone microarchitecture, measured by HR-pQCT, in osteoporosis subjects [33]; therefore, we postulated that FGF 23 and α-klotho are associated with TBS. However, we did not find any association between TBS and FGF 23 or α-klotho in the present study. We performed multivariable regression analysis to determine the factors associated with ln FGF 23, adjusting for age, dialysis vintage, DM, CVE, albumin, calcium, phosphate, intact PTH, ln α-klotho, handgrip strength, and phosphate binder dose; as a result, calcium, phosphate, and intact PTH showed significant positive correlation with FGF 23 (data not shown). These results indicate that there are several confounding factors between FGF 23 and bone. Previous studies suggest that FGF 23 affects bone both directly [34] and indirectly through a FGF 23-bone-kidney axis [35]. Bone mineral metabolism is a complex process mediated by multi-organ interactions; therefore, these various confounders, such as phosphate, calcium, vitamin D, PTH, and α-klotho may obscure the relationship between FGF 23 and bone microarchitecture. A prior study also reported that TBS was not associated with FGF 23 [17].

Because TBS is a static test, it cannot measure dynamic bone turnover status or distinguish the types of renal osteodystrophy. The discrimination of bone turnover status is important because treatment varies according to changes in bone remodeling. KDIGO guidelines suggest that PTH or bone-specific ALP can be used to evaluate bone turnover [1]. A recent study reported that TBS is inversely related to PTH and ALP [18]. In the present study, only PTH showed a significant positive correlation with TBS in univariate analysis. Several studies have revealed that hyperparathyroidism increases cortical bone loss while causing anabolic effects on trabecular bone [36, 37]. These findings, and confounders such as age or nutritional state, may explain this positive correlation; however, after adjustment for various relevant parameters, the relationship disappeared. On a theoretical basis, continuous hyperparathyroidism accelerates bone resorption, resulting in decreased bone mass and impaired bone microarchitecture. In the case of BMD, the relationship with PTH is either non-significant or significant but inversely correlated [1]. To date, few studies have evaluated TBS and bone turnover markers (except PTH or ALP) together in CKD populations. Currently, a prospective observation study of TBS and bone remodeling markers as fracture risk factors is being carried out with 206 non-dialysis CKD stage 4–5 and kidney transplanted patients (NCT03356522).

Recent data showed that both PPI and warfarin are associated with increased risk of fracture, cardiovascular disease, and mortality in dialysis patients [38–40]. PPI use is associated with mineral and vitamin deficiency, including magnesium, calcium, or vitamin B12, which contribute to bone health. Particularly, hypomagnesemia is related to vascular calcification and cardiovascular disease, which in turn increases mortality. Vitamin K deficiency caused by warfarin may promotes vascular calcification process and increased fracture risk. So we conducted statistical analysis to determine if these drug use and incident fracture, incident CVE, mortality, or TBS were related (see Additional file 1). As a result, in our study, PPI and warfarin use were not related to clinical outcomes and TBS. Because small numbers of patients were taking PPI or warfarin, we thought that this factor affected to the results.

Our study has several limitations. First, it was a single-center study with a small sample size and the follow-up period was relatively short. Second, our mean TBS value was 1.44, which was higher than previously reported in ESRD subjects. Generally, TBS ≥ 1.31 is considered homogeneously textured bone and TBS ≤ 1.23 indicates less well-textured bone. In a previous study, the mean TBS value of ESRD population was 1.11–1.34 [16–20]. It is likely that overall TBS was biased toward high values in the present study. However, because it was measured in a single center with the same methods, we do not think that possible bias should affect our between-group analyses. Third, bone turn-over markers related to CKD-MBD, other than PTH and ALP, inflammatory markers or vitamin D, were not available, and we did not perform bone biopsies. Lastly, as all our subjects were Korean, the results may not be applicable to other ethnicities.

BMD may be falsely elevated in CKD due to vascular or tissue calcification and/or degenerative changes of the lumbar spine, which are prevalent in CKD. TBS may hold certain advantages over BMD in assessing mineral-bone disease in this unique population, as it is not impacted by these conditions. In addition, TBS can detect microarchitectural changes which BMD cannot adequately assess. However, to date, TBS has only been used as a complement to other tools, such as FRAX or BMD, to improve fracture prediction in clinical practice and has not been validated for isolated use. Also, TBS cannot measure ongoing bone turnover status, which limits its usefulness for guiding therapeutic decisions. It is possible that studies of serial TBS measurements with bone marker and HR-qQCT or bone biopsy may provide information about its use in therapeutic strategies. And hopefully, after several data accumulations, TBS may able to replace these expensive and/or invasive methods. Our study has shown that TBS may provide valuable information about adverse outcomes related to CKD-MBD in HD patients, and TBS could be a replacement diagnostic tool for more expensive traditional methods. Further studies are needed to thoroughly determine the prognostic and therapeutic utility of TBS for HD patients.

## Conclusion

In HD patients, age and prevalent CVE were independently associated with TBS, and mortality and incident fracture were significantly higher in the lower TBS group. These findings suggest that TBS may be useful for predicting adverse outcomes related to CKD-MBD, and not only fractures but also CVE or morality. Further study is needed to fully elucidate the clinical utility of TBS in HD patients.

## Supplementary information

**Additional file 1.** Clinical outcomes and TBS according to medication use (PPI or warfarin).

## Data Availability

The datasets used and/or analyzed during the current study are available from the corresponding author on reasonable request.

## Abbreviations

ALP: Alkaline phosphatase
BIA: Bioelectrical impedance analysis
BMD: Bone mineral density
BMI: Body mass index
CAC: Coronary artery calcification
CAD: Coronary artery disease
CKD: Chronic kidney disease
CKD-MBD: Chronic kidney disease-mineral and bone disorder
CVE: Cardiovascular events
DM: Diabetes mellitus
DXA: Dural-energy x-ray absorptiometry
ECLIA: Electrochemiluminescence immunoassay
ESRD: End stage renal disease
FGF-23: Fibroblast growth factor 23
HR-pQCT: High-resolution peripheral quantitative computed tomography
HTN: Hypertension
KDIGO: Kidney Disease Improving Global Outcomes
MRI: Magnetic resonance imaging
PAOD: Peripheral arterial occlusive disease
PG-SGA: Patient-generated subjective global assessment
PPI: Proton pump inhibitor
PTH: Parathyroid hormone
SD: Standard deviation
TBS: Trabecular bone score
VC: Vascular calcification

## References

[1] Kidney Disease: Improving Global Outcomes (KDIGO) CKD-MBD Work Group. KDIGO Clinical practice guideline for the diagnosis, evaluation, prevention, and treatment of Chronic Kidney Disease-Mineral and Bone Disorder (CKD-MBD). Kidney Int. 2009;76(suppl 113):S1–130. 10.1038/ki.2009.188 19644521

[2] Foley RN, Parfrey PS, Sarnak MJ (1998). Epidemiology of cardiovascular disease in chronic renal disease. J Am Soc Nephrol.

[3] Alem AM, Sherrard DJ, Gillen DL, Weiss NS, Beresford SA, Heckbert SR (2000). Increased risk of hip fracture among patients with end-stage renal disease. Kidney Int.

[4] Fusaro M, Tripepi G, Noale M, Vajente N, Plebani M, Zaninotto M (2013). High prevalence of vertebral fractures assessed by quantitative morphometry in hemodialysis patients, strongly associated with vascular calcifications. Calcif Tissue Int.

[5] Ketteler M, Block GA, Evenepoel P, Fukagawa M, Herzog CA, McCann L (2017). Executive summary of the 2017 KDIGO chronic kidney Disease-mineral and bone disorder (CKD-MBD) guideline update: what's changed and why it matters. Kidney Int.

[6] Jamal SA, Chase C, Goh YI, Richardson R, Hawker GA (2002). Bone density and heel ultrasound testing do not identify patients with dialysis-dependent renal failure who have had fractures. Am J Kidney Dis.

[7] Negri AL, Barone R, Quiroga MA, Bravo M, Marino A, Fradinger E (2004). Bone mineral density: serum markers of bone turnover and their relationships in peritoneal dialysis. Perit Dial Int.

[8] Pimentel A, Urena-Torres P, Zillikens MC, Bover J, Cohen-Solal M (2017). Fractures in patients with CKD-diagnosis, treatment, and prevention: a review by members of the European calcified tissue society and the European renal Association of Nephrology Dialysis and Transplantation. Kidney Int.

[9] Martineau P, Leslie WD (2017). Trabecular bone score (TBS): method and applications. Bone..

[10] Silva BC, Leslie WD, Resch H, Lamy O, Lesnyak O, Binkley N (2014). Trabecular bone score: a noninvasive analytical method based upon the DXA image. J Bone Miner Res.

[11] Naylor KL, Prior J, Garg AX, Berger C, Langsetmo L, Adachi JD (2016). Trabecular bone score and incident fragility fracture risk in adults with reduced kidney function. Clin J Am Soc Nephrol.

[12] Ramalho J, Marques IDB, Hans D, Dempster D, Zhou H, Patel P (2018). The trabecular bone score: relationships with trabecular and cortical microarchitecture measured by HR-pQCT and histomorphometry in patients with chronic kidney disease. Bone..

[13] Hans D, Barthe N, Boutroy S, Pothuaud L, Winzenrieth R, Krieg MA (2011). Correlations between trabecular bone score, measured using anteroposterior dual-energy X-ray absorptiometry acquisition, and 3-dimensional parameters of bone microarchitecture: an experimental study on human cadaver vertebrae. J Clin Densitom.

[14] Roux JP, Wegrzyn J, Boutroy S, Bouxsein ML, Hans D, Chapurlat R (2013). The predictive value of trabecular bone score (TBS) on whole lumbar vertebrae mechanics: an ex vivo study. Osteoporos Int.

[15] Pothuaud L, Carceller P, Hans D (2008). Correlations between grey-level variations in 2D projection images (TBS) and 3D microarchitecture: applications in the study of human trabecular bone microarchitecture. Bone..

[16] Brunerova L, Ronova P, Veresova J, Beranova P, Potoekova J, Kasalicky P (2016). Osteoporosis and impaired trabecular bone score in hemodialysis patients. Kidney Blood Press Res.

[17] Yavropoulou MP, Vaios V, Pikilidou M, Chryssogonidis I, Sachinidou M, Tournis S (2017). Bone quality assessment as measured by trabecular bone score in patients with end-stage renal Disease on Dialysis. J Clin Densitom.

[18] Aleksova J, Kurniawan S, Elder GJ (2018). The trabecular bone score is associated with bone mineral density, markers of bone turnover and prevalent fracture in patients with end stage kidney disease. Osteoporos Int.

[19] Dusceac R, Niculescu DA, Dobre R, Dragne MC, Tacu C, Peride I (2018). Chronic hemodialysis is associated with lower trabecular bone score, independent of bone mineral density: a case-control study. Arch Osteoporos.

[20] Yoon HE, Kim Y, Shin SJ, Hong YS, Kang KY (2019). Factors associated with low trabecular bone scores in patients with end-stage kidney disease. J Bone Miner Metab.

[21] London GM (2009). Bone-vascular axis in chronic kidney disease: a reality?. Clin J Am Soc Nephrol.

[22] Cejka D, Weber M, Diarra D, Reiter T, Kainberger F, Haas M (2014). Inverse association between bone microarchitecture assessed by HR-pQCT and coronary artery calcification in patients with end-stage renal disease. Bone..

[23] Barreto DV, Barreto Fde C, Carvalho AB, Cuppari L, Draibe SA, Dalboni MA (2008). Association of changes in bone remodeling and coronary calcification in hemodialysis patients: a prospective study. Am J Kidney Dis.

[24] Aleksova J, Kurniawan S, Vucak-Dzumhur M, Kerr P, Ebeling PR, Milat F (2018). Aortic vascular calcification is inversely associated with the trabecular bone score in patients receiving dialysis. Bone..

[25] Lopez-Soto PJ, De Giorgi A, Senno E, Tiseo R, Ferraresi A, Canella C (2015). Renal disease and accidental falls: a review of published evidence. BMC Nephrol.

[26] Abdel-Rahman EM, Turgut F, Turkmen K, Balogun RA (2011). Falls in elderly hemodialysis patients. QJM..

[27] Reginster JY, Beaudart C, Buckinx F, Bruyere O (2016). Osteoporosis and sarcopenia: two diseases or one?. Curr Opin Clin Nutr Metab Care.

[28] Locquet M, Beaudart C, Bruyere O, Kanis JA, Delandsheere L, Reginster JY (2018). Bone health assessment in older people with or without muscle health impairment. Osteoporos Int.

[29] Olauson H, Vervloet MG, Cozzolino M, Massy ZA, Urena Torres P, Larsson TE (2014). New insights into the FGF23-Klotho axis. Semin Nephrol.

[30] Hu MC, Kuro-o M, Moe OW (2013). Klotho and chronic kidney disease. Contrib Nephrol.

[31] Urena Torres P, Friedlander G, de Vernejoul MC, Silve C, Prie D (2008). Bone mass does not correlate with the serum fibroblast growth factor 23 in hemodialysis patients. Kidney Int.

[32] Zheng S, Chen Y, Zheng Y, Zhou Z, Li Z (2018). Correlation of serum levels of fibroblast growth factor 23 and Klotho protein levels with bone mineral density in maintenance hemodialysis patients. Eur J Med Res.

[33] Rupp T, Butscheidt S, Vettorazzi E, Oheim R, Barvencik F, Amling M (2019). High FGF23 levels are associated with impaired trabecular bone microarchitecture in patients with osteoporosis. Osteoporos Int.

[34] Wang H, Yoshiko Y, Yamamoto R, Minamizaki T, Kozai K, Tanne K (2008). Overexpression of fibroblast growth factor 23 suppresses osteoblast differentiation and matrix mineralization in vitro. J Bone Miner Res.

[35] Martin A, David V, Quarles LD (2012). Regulation and function of the FGF23/klotho endocrine pathways. Physiol Rev.

[36] Duan Y, De Luca V, Seeman E (1999). Parathyroid hormone deficiency and excess: similar effects on trabecular bone but differing effects on cortical bone. J Clin Endocrinol Metab.

[37] Parisien M, Silverberg SJ, Shane E, de la Cruz L, Lindsay R, Bilezikian JP (1990). The histomorphometry of bone in primary hyperparathyroidism: preservation of cancellous bone structure. J Clin Endocrinol Metab.

[38] Fusaro M, D'Arrigo G, Pitino A, Iervasi G, Tentori F, Robinson B (2019). Increased risk of bone fractures in hemodialysis patients treated with proton pump inhibitors in real world: results from the Dialysis outcomes and practice patterns study (DOPPS). J Bone Miner Res.

[39] de Francisco ALM, Varas J, Ramos R, Merello JI, Canaud B, Stuard S (2018). Proton pump inhibitor usage and the risk of mortality in hemodialysis patients. Kidney Int Rep.

[40] Fusaro M, Tripepi G, Noale M, Plebani M, Zaninotto M, Piccoli A (2015). Prevalence of vertebral fractures, vascular calcifications, and mortality in warfarin treated hemodialysis patients. Curr Vasc Pharmacol.

