# Supplementary material for: Trabecular bone score may indicate chronic kidney disease-mineral and bone disorder (CKD-MBD) phenotypes in hemodialysis patients: a prospective observational study
Source: BMC Nephrol. 2020 Jul 25;21:299. doi: 10.1186/s12882-020-01944-0 (PMC7382149; doi:10.1186/s12882-020-01944-0)
Supplement: Supplementary file 1 — Additional file 1. Clinical outcomes and TBS according to medication use (PPI or warfarin). [file 12882_2020_1944_MOESM1_ESM.docx]

Additional file 1. Clinical outcomes and TBS according to medication use (PPI or warfarin)

|  |  | PPI  (N=11) | No PPI  (N=46) | p-value | Warfarin  (N=1) | No warfarin  (N=56) | p-value |
| --- | --- | --- | --- | --- | --- | --- | --- |
| Fracture | Yes (N=7) | 0 (0%) | 7 (12.3%) | 0.325^*^ | 1 (1.8%) | 6 (10.5%) | 0.123^*^ |
|  | No (N=50) | 11 (19.3%) | 39 (68.4%) |  | 0 (0%) | 50 (87.7%) |  |
| CVE | Yes (N=6) | 1 (1.8%) | 5 (8.8%) | 1.000^*^ | 0 (0%) | 6 (10.5%) | 1.000^*^ |
|  | No (N=51) | 10 (17.5%) | 41 (71.9%) |  | 1 (1.8%) | 50 (87.7%) |  |
| Mortality | Yes (N=4) | 1 (1.8%) | 3 (5.3%) | 1.000^*^ | 0 (0%) | 4 (7.0%) | 1.000^*^ |
|  | No (N=53) | 10 (17.5%) | 43 (75.4%) |  | 1 (1.8%) | 52 (91.2%) |  |
| TBS | | 1.40±0.10 | 1.45±0.10 | 0.144^†^ | 1.32 | 1.44±0.10 | 0.224^‡^ |

^*^ *p*-value by Fisher’s exact test

^†^ *p*-value by t-test

^‡^ *p*-value by Mann–Whitney *U* test

Abbreviations: PPI, Proton pump inhibition; CVE, Cardiovascular events; TBS, Trabecular bone score
